# Supplementary material for: Geographic distribution modeling and taxonomy of Stephadiscus lyratus (Cothouny in Gould, 1846) (Charopidae) reveal potential distributional areas of the species along the Patagonian Forests
Source: PeerJ. 2021 Jul 5;9:e11614. doi: 10.7717/peerj.11614 (PMC8265385; doi:10.7717/peerj.11614)
Supplement: Supplemental Information 1 [file peerj-09-11614-s001.docx]

**Table S1**

| **Collection Numbers** | **Locality** | **Country** | **Latitud** | **Longitud** | **Collection year** | **Bibliographic/Museum Source** |
| --- | --- | --- | --- | --- | --- | --- |
| MACN-In 9997 | Santa Ines Cape, Rio Grande, Tierra del Fuego | Argentina | -54.134116 | -67.09908 | 1921 | Miquel & Araya (2013) |
| MACN-In 12384 | Rio Las Minas, Punta Arenas | Chile | -53.143489 | -70.983491 | 1921 | Miquel & Araya (2013) |
| MACN-In 12754 | Rio Grande, Tierra del Fuego | Argentina | -53.80645 | -67.795728 | 1921 | Miquel & Araya (2013) |
| MACN-In 21168 | Ushuaia, Tierra del Fuego | Argentina | -54.825324 | -68.361069 | 1933 | Miquel & Araya (2013) |
| MACN-In 22173 | Puerto San Juan, Isla de los estados | Argentina | -54.735508 | -63.86752 | 1933 | Miquel & Araya (2013) |
| MACN-In 22525 | Puerto Cook, Isla de los Estados | Argentina | -54.766858 | -64.049921 | 1935 | Miquel & Araya (2013) |
| MACN-In 27226 | Lapataia, Tierra del Fuego | Argentina | -54.831997 | -68.555016 | 1965 | Miquel & Araya (2013) |
| MACN-In 27464 | Isla Observatorio, Archipielago Año Nuevo | Argentina | -54.665897 | -64.135028 | 1971 | Miquel & Araya (2013) |
| MACN-In 29351 | En frente a Paso Garibaldi | Argentina | -54.683306 | -67.820304 | 1971 | Miquel & Araya (2013) |
| MACN-In 31759 | Seno Ultima Esperanza, Magallanes | Chile | -51.546478 | -72886545 | 1977 | Miquel & Araya (2013) |
| MACN-In 36084 | Isla Redonda, Canal de Beagle | Argentina | -54.864836 | -68.477265 | 1999 | Miquel & Araya (2013) |
| MACN-In 36088 | Laguna Victoria, Tierra del Fuego | Argentina | -54.77621 | -67.701475 | 1985 | Miquel & Araya (2013) |
| MACN-In 31734 | Glaciar del Cerro Balmaceda | Chile | ̶ | ̶ | 1977 | Miquel & Cadiz Lorca (2008) |
| MACN-In 9997 | Cabo Santa Ines, Tierra del Fuego | Argentina | ̶ | ̶ | 1921 | Museum material |
| MACN-In 27277 | Isla de los Estados | Argentina | -54.76268 | -64.271468 | 1967 | Museum material |
| MACN-In 27463 | Bahia del Buen Suceso, Tierra del Fuego | Argentina | -54.884451 | -65.462746 | 1971 | Museum material |
| MACN-In 29327 | Villa Carbajal | Argentina | ̶ | ̶ | ͞͞ | Hylton Scott (1970) |
| MACN-In 30102 | Ancon sin salida | Chile | ̶ | ̶ | 1977 | Museum material |
| MACN-In 32846 | Lago Fagnano, Tierra del Fuego | Argentina | -54.610919 | -67.40515 | 1965 | Museum material |
| MACN-In 33049 | Lago Fagnano, Tierra del Fuego | Argentina | -54.610919 | -67.40515 | 1966 | Museum material |
| MACN-In 33660 | Ruta complementaria, Tierra del Fuego | Argentina | ̶ | ̶ | 1984 | Museum material |
| MACN-In 36065 | Isla Navarino | Chile | -54.944385 | -67.627622 |  | Museum material |
| MACN-In 31731 | Close to Puerto Natales | Chile | -51.8333 | -73.0833 | 1979 | Museum material |
| MACN-In 24464 | Isla Alferez Gofre | Argentina | ̶ | ̶ | 1971 | Hylton Scott (1972) |
| MLP 4384-1 | Vancouver Bay, Isla de los Estados | Argentina | -54.781507 | -64.063391 | ̶ | Miquel & Araya (2013) |
| MLP 10.482 | Port William, Navarino Island | Chile | -54.939556 | -67.62067 | ̶ | Miquel & Araya (2013) |
| MLP 10485 | 40km between Natales and Magallanes | Chile | -51.997681 | -72.09694 | ̶ | Miquel & Araya (2013) |
| MLP 10.486 | Buen Suceso Bay, Tierra del Fuego | Argentina | -54.884451 | -65.462746 | ̶ | Miquel & Araya (2013) |
| MLP 10.488 | Douglas River, close to Murray Chanell, Navarino | Chile | -55.173192 | -68.036088 | ̶ | Miquel & Araya (2013) |
| MLP10.492 | Picton Island | Chile | -55.611563 | -66.955083 | ̶ | Miquel & Araya (2013) |
| MLP 10.500 | Fagnano Lake, Tierra del Fuego | Argentina | -54.557981 | -67.23841 | ̶ | Miquel & Araya (2013) |
| IBN 944 | Sendero Hito XXIV | Argentina | -54.8276 | -68.56195 | 2018 | This manuscript |
| IBN | Sendero Hito XXIV | Argentina | -54.80866 | -68.59528 | 2018 | This manuscript |
| IBN | Esmeralda Lake, Tierra del Fuego | Argentina | -54.70249 | -68.12815 | 2018 | This manuscript |
| IBN | Piloto River, Pampa Alta,Tierra del Fuego | Argentina | -54.83873 | -68.48845 | 2019 | This manuscript |
| IBN 951 | Hito XXIV trail, NPTF | Argentina | -54.8151 | -68.58623 | 2018 | This manuscript |
| IBN | Hito XXIV trail, NPTF | Argentina | -54.82302 | -68.56677 | 2019 | This manuscript |
| IBN | Hito XXIV trail, NPTF | Argentina | -54.82416 | -68.56424 | 2019 | This manuscript |
| IBN 955 | Hito XXIV trail, NPTF | Argentina | -54.824295 | -68.564407 | 2019 | This manuscript |
| IBN | Glacial Martial trail, Tierra del Fuego | Argentina | -54.79644 | -68.37686 | 2019 | This manuscript |
| IBN 958 | Nunatak Trail, Tierra del Fuego | Argentina | -54.70812 | -68.10475 | 2019 | This manuscript |
| IBN 960 | Cerro Castor,Tierra del Fuego | Argentina | -54.71884 | -68.03739 | 2019 | This manuscript |
| IBN | Pampa Alta trail, PNTF | Argentina | -54.73988 | -68.30956 | 2019 | This manuscript |
| IBN | Estancia Moat, Tierra del Fuego | Argentina | -54.97743 | -66.72418 | 2019 | This manuscript |
| IBN 961 | Estancia Moat, Tierra del Fuego | Argentina | -54.97126 | -66.73016 | 2019 | This manuscript |
| IBN | Estancia Moat, Tierra del Fuego | Argentina | -54.96908 | -66.73074 | 2019 | This manuscript |
| IBN | Sendero Hito XXIV, NPTF | Argentina | -54.82427 | -68.56436 | 2019 | This manuscript |
| IBN | Sendero Hito XXIV, NPTF | Argentina | -54.82417 | -68.56478 | 2019 | This manuscript |
| IBN | Ushuaia, Tierra del Fuego | Argentina | -54.81992 | -68 | 2019 | This manuscript |
| IBN | Playa Larga trail, close to Ushuaia, Tierra del Fuego | Argentina | -54.82031 | -68.16084 | 2019 | This manuscript |
| IBN | Bahia Lapataia, Tierra del Fuego | Argentina | -54.8575 | -68.57053 | 2019 | This manuscript |
| Without collection number | Isla de los Estados, Puerto Cook | Argentina | -54.76268 | -64.271468 | ̶ | Hylton Scott (1970) |
| Without collection number | Puerto Vancouver, Isla de los Estados | Argentina | ̶ | ̶ | ̶ | Hylton Scott (1970) |
| Without collection number | Lago Argentino, Santa Cruz | Argentina | -50.467264 | -73.013603 | ̶ | Hylton Scott (1970) |
| Without collection number | Isla Navarino | Chile | -54.944385 | -67.627622 | ̶ | Hylton Scott (1970) |
| Without collection number | Bahia Aguirre, Tierra del Fuego | Argentina | ̶ | ̶ | ̶ | Hylton Scott (1970) |
| Without collection number | Bahia Encerrada, Tierra del Fuego | Argentina | -54.814484 | -68.316254 | ̶ | Museum material |
| Without collection number | Bahia del Buen Suceso, Tierra del Fuego | Argentina | -54.884451 | -65.462746 | ̶ | Hylton Scott (1972) |
| Without collection number | Bahia Liberty | Argentina | ̶ | ̶ | ̶ | Hylton Scott (1972) |
