# Supplementary material for: Geographic distribution modeling and taxonomy of Stephadiscus lyratus (Cothouny in Gould, 1846) (Charopidae) reveal potential distributional areas of the species along the Patagonian Forests
Source: PeerJ. 2021 Jul 5;9:e11614. doi: 10.7717/peerj.11614 (PMC8265385; doi:10.7717/peerj.11614)
Supplement: Supplemental Information 2 — The gray column shows the low number of specimens on which the original descriptions were based. [file peerj-09-11614-s002.docx]

| **Species** | **Type locality** | **Eco/sub-eregion distribution** | **Type material** | **DM (mm)** | **Dm (mm)** | **H (mm)** | **General shell shape** | **Protoconch sulpture** | **Teleoconch sculpture** | **Aperture shape** | **Umbilicus** | **N° specimens original description** | **Source** |
| --- | --- | --- | --- | --- | --- | --- | --- | --- | --- | --- | --- | --- | --- |
| ***Stephadiscus lyratus*** (Couthouny in Gould, 1846) | Orange Harbor, Tierra del Fuego | Magellanic Subpolar forests | MCZ 88297 | 5.5* 4,24-5,04** | 4* 3,44 - 4,48** | 2.2* 2,28-2,43** | Discoidal, depresed spire. Not planispiral as apex elevated. | 35 - 40 axial, smooth, elevated ribs separated at regular intervals. Spaces between ribs with thinner axial costulae. | Major axial ribs, with 5 to 7 micro radial costulas in between major ribs, nodules at regular intervals supporting ribs | Circular aperture, not descendent, with sharp peristome | Perspective, narrow, 1/3 or slightly less of body whorl diameter | Not reported | Gould (1846); *Hylton Scott (1973); Hylton Scott (1970); **this study |
| ***Stephadiscus mirabilis*** (Hylton Scott, 1968) | Isla Victoria, Nahuel Huapi, Argentina | Valdivian Temperate Forest | MLP  (type)-MACN 21755 | 4.8 | 4.2 | 2 | Orbicular depressed, flattened | "radial striation" | Regular and spaced lamellar ribs with spiral ridges | Almost vertical subcircular aperture |  | 3 (1 type, 2 dissected)+8 (paratypes) | Hylton Scott (1968) |
| ***Stephadiscus celinae*** (Hylton Scott, 1969) | Villa La Angostura, Peninsula Quetrihue, Neuquen, Argentina | Valdivian Temperate Forest | MACN | 3 | 2.65 | 1.7 | Orbicular, vaulted, not planispiral | "deep radial striation" | Axial ribs separated. Inter space with growth lines | Subcircular aperture | Widly open, 1/3 of major diameter | 1 paratype+ 2 juvenile specimens | Hylton Scott (1969) |
| ***Stephadiscus* *perversus*** (Hylton Scott, 1969 | Puerto Blest, Nahuel Huapi, Argentina | Valdivian Temperate Forest | ? | 2.8 | 2.5 | 1.5 | Discoidal, with dorsal side sub flat | "manifest radial sculpture which is continued in the post embryonic whorls in the form of thin ribs" | Thin ribs separated by spaces wider than the ribs or in other whorls, spaces equal to the width of the rib | Vertical aperture, higher than wide | Less than 1/3 of the major diameter | 2 specimens (1 juvenil) | Hylton Scott (1969) |
| ***Stephadiscus rumbolli*** (Hylton Scott, 1973) | Puerto San Carlos, Malvinas, Argentina | Magellanic Subpolar forests. Patagonian Steppe | Holotype MLP MACN 27815 (paratype) | 1.7 | 1.4 | 1 | Orbicular with flatted spire,apex immersed | "thin, regular and closely spaced radial ribs" (costulas radiales delgadas, regular y estrechamente espaciadas) | Axial ribs separated. Inter space with growth lines | Aperture taller than wide | Narrow, less than 1/3 of the major shell diameter | 2 specimens (both juveniles) | Hylton Scott (1973) |
| ***Stephadiscus stuardoi*** Miquel & Barker, 2009 | Hualpén Botanical Park, Concepcion, Chile | Valdivian Temperate Forest | MNHNCL 6655 (Holotype) MACN-In 36142 (Topotype) | 2.0-2.01 | ? | 0.85 | Discoidal, almost planispiral with apex slightly elevated | "closely spaced axial ribs lacking spiral threads" | Slightly sigmoideal axial ribs and weak spiral threads |  | 0.25 of shell diameter, scarcely perspective | 2 specimens | Mique & Barker (2009) |
| ***Stephanoda antarctica*** Hylton Scott, 1973 | Puerto San Carlos, Malvinas, Argentina | Patagonian Steppe | MACN 27820 | 2.8 | 2.3 | 1.6 | Helicoidal with low spire | Net radial striation ending at the first post-embryonic radial rib | Radial ribs with wide space between them | Circular |  | Single especimen | Hylton Scott (1973) |
| ***Stephanoda dissimilis*** (d´Orbigny, 1835) | Concepcion, Chile | Valdivian Temperate Forest | NMNH (London) | 9 | ? | 5 | Suborbicular, depressed, obtuse spire | Transverse and longitudinal striae |  | Semilunar |  | Not reported | d¨Orbigny (1835) |
| ***Stephacharopa distincta*** (Hylton Scott, 1970) | Zona Rio Foyel, Rio Negro, Argentina | Valdivian Temperate Forest | MACN-In | 2.1 | 1.8 | 1.3 | Orbicular, slightly vaulted | "smooth on the first whorl, with fine radial striae on the second whorl" | Radial ribs, spaced slightly wider than ribs | Kidney-shaped, vertical | Less than 1/3 of the shell diameter | 1+1 juvenil | Hylton Scott (1970) |
| ***Stephacharopa testalba*** (Hylton Scott, 1970) | Zona Lago Mascardi, Nahuel Huapi, Argentina | Valdivian Temperate Forest | MLP 10.511 | 2.65 | 2.3 | 1.35 | Orbicular, slightly vaulted | "net radial sculpture" diferent from teleoconch sculpture | Radial ribs, thin and low, equidistant separated by spaces about 3 times their width | Subcircular, vertical | 1/4 of the shell diameter | Not reported | Hylton Scott (1970) |
| ***Stephacharopa calderaensis*** Miquel & Araya, 2013 | Quebrada del Leon, about 15km east of Caldera, Copiapo, Atacama, Chile | 1 | MNHNCL 7593 (Holotype) MACN-In 39022 (Paratype) | 2.1 | ? | 1.05 | Orbicular, discoidal | "thin, smooth radial ribs"diferent from teleoconch sculpture | Sculptured with 90-95 prominent, wide, prosocline, nodulose radial ribs | Circular, descendent | Widely perspective of 0.3 of shell diameter | More than 18 specimens | Miquel & Araya (2013) |
