# Supplementary material for: Geographic distribution modeling and taxonomy of Stephadiscus lyratus (Cothouny in Gould, 1846) (Charopidae) reveal potential distributional areas of the species along the Patagonian Forests
Source: PeerJ. 2021 Jul 5;9:e11614. doi: 10.7717/peerj.11614 (PMC8265385; doi:10.7717/peerj.11614)
Supplement: Supplemental Information 3 [file peerj-09-11614-s003.docx]

**Table S3. Model performance of candidate models acording regularization multiplier (RM) and feature classes (FC, l: linear, q: quadratic, p: product).** The selection criteria were partial ROC (pROC), omission rate and AIC.

| **RM** | **FC** | **Mean_AUC_ratio** | **pval pROC** | **Omission rate 5%** | **AICc** | **Delta AICc** | **Weight AICc** | **Number of parameters** |
| --- | --- | --- | --- | --- | --- | --- | --- | --- |
| 0.1 | qp | 1.45 | 0 | 0.29 | 806.93 | 9.58 | 0.00 | 14 |
| 0.1 | lq | 1.51 | 0 | 0.29 | 802.36 | 5.01 | 0.00 | 12 |
| 0.1 | l | 1.42 | 0 | 0.29 | 812.87 | 15.52 | 0.00 | 6 |
| 0.1 | q | 1.50 | 0 | 0.29 | 806.07 | 8.72 | 0.00 | 7 |
| 0.1 | p | 1.49 | 0 | 0.29 | 825.94 | 28.59 | 0.00 | 14 |
| 0.1 | lqp | 1.43 | 0 | 0.29 | 797.35 | 0.00 | 0.05 | 12 |
| 0.5 | qp | 1.47 | 0 | 0.29 | 803.50 | 6.15 | 0.00 | 7 |
| 0.5 | lq | 1.45 | 0 | 0.29 | 805.44 | 8.09 | 0.00 | 9 |
| 0.5 | l | 1.40 | 0 | 0.29 | 813.77 | 16.42 | 0.00 | 6 |
| 0.5 | q | 1.46 | 0 | 0.29 | 807.61 | 10.26 | 0.00 | 7 |
| 0.5 | p | 1.48 | 0 | 0.29 | 807.47 | 10.12 | 0.00 | 7 |
| 0.5 | lqp | 1.44 | 0 | 0.29 | 803.47 | 6.12 | 0.00 | 7 |
| 1 | qp | 1.50 | 0 | 0.29 | 804.90 | 7.55 | 0.00 | 6 |
| 1 | lq | 1.50 | 0 | 0.29 | 808.07 | 10.72 | 0.00 | 7 |
| 1 | l | 1.41 | 0 | 0.29 | 811.50 | 14.15 | 0.00 | 5 |
| 1 | q | 1.46 | 0 | 0.29 | 806.54 | 9.19 | 0.00 | 6 |
| 1 | p | 1.44 | 0 | 0.29 | 809.95 | 12.60 | 0.00 | 6 |
| 1 | lqp | 1.44 | 0 | 0.29 | 804.90 | 7.55 | 0.00 | 6 |
| 2 | qp | 1.46 | 0 | 0.29 | 808.57 | 11.22 | 0.00 | 5 |
| 2 | lq | 1.47 | 0 | 0.29 | 806.76 | 9.41 | 0.00 | 5 |
| 2 | l | 1.44 | 0 | 0.29 | 809.42 | 12.07 | 0.00 | 4 |
| 2 | q | 1.43 | 0 | 0.29 | 804.15 | 6.80 | 0.01 | 4 |
| 2 | p | 1.46 | 0 | 0.29 | 817.39 | 20.04 | 0.00 | 5 |
| 2 | lqp | 1.49 | 0 | 0.29 | 808.57 | 11.22 | 0.00 | 5 |
