# Supplementary material for: Geographic distribution modeling and taxonomy of Stephadiscus lyratus (Cothouny in Gould, 1846) (Charopidae) reveal potential distributional areas of the species along the Patagonian Forests
Source: PeerJ. 2021 Jul 5;9:e11614. doi: 10.7717/peerj.11614 (PMC8265385; doi:10.7717/peerj.11614)
Supplement: Supplemental Information 4 [file peerj-09-11614-s004.docx]

**Table S4. Settings and model performance of the final model.** RM (regularization multiplier), FC (feature clases, l: linear, q: quadratic, p: product), pROC (partial ROC), omission rate and AIC.

| **RM** | **FC** | **Mean_AUC_ratio** | **pval pROC** | **Omission rate 5%** | **AICc** | **Delta AICc** | **Weight AICc** | **Number of parameters** |
| --- | --- | --- | --- | --- | --- | --- | --- | --- |
| 0.1 | lqp | 1.43 | 0 | 0.29 | 797.35 | 0.00 | 0.05 | 12 |
