# Supplementary figures and images for: Geographic distribution modeling and taxonomy of Stephadiscus lyratus (Cothouny in Gould, 1846) (Charopidae) reveal potential distributional areas of the species along the Patagonian Forests
Source: PeerJ. 2021 Jul 5;9:e11614. doi: 10.7717/peerj.11614 (PMC8265385; doi:10.7717/peerj.11614)

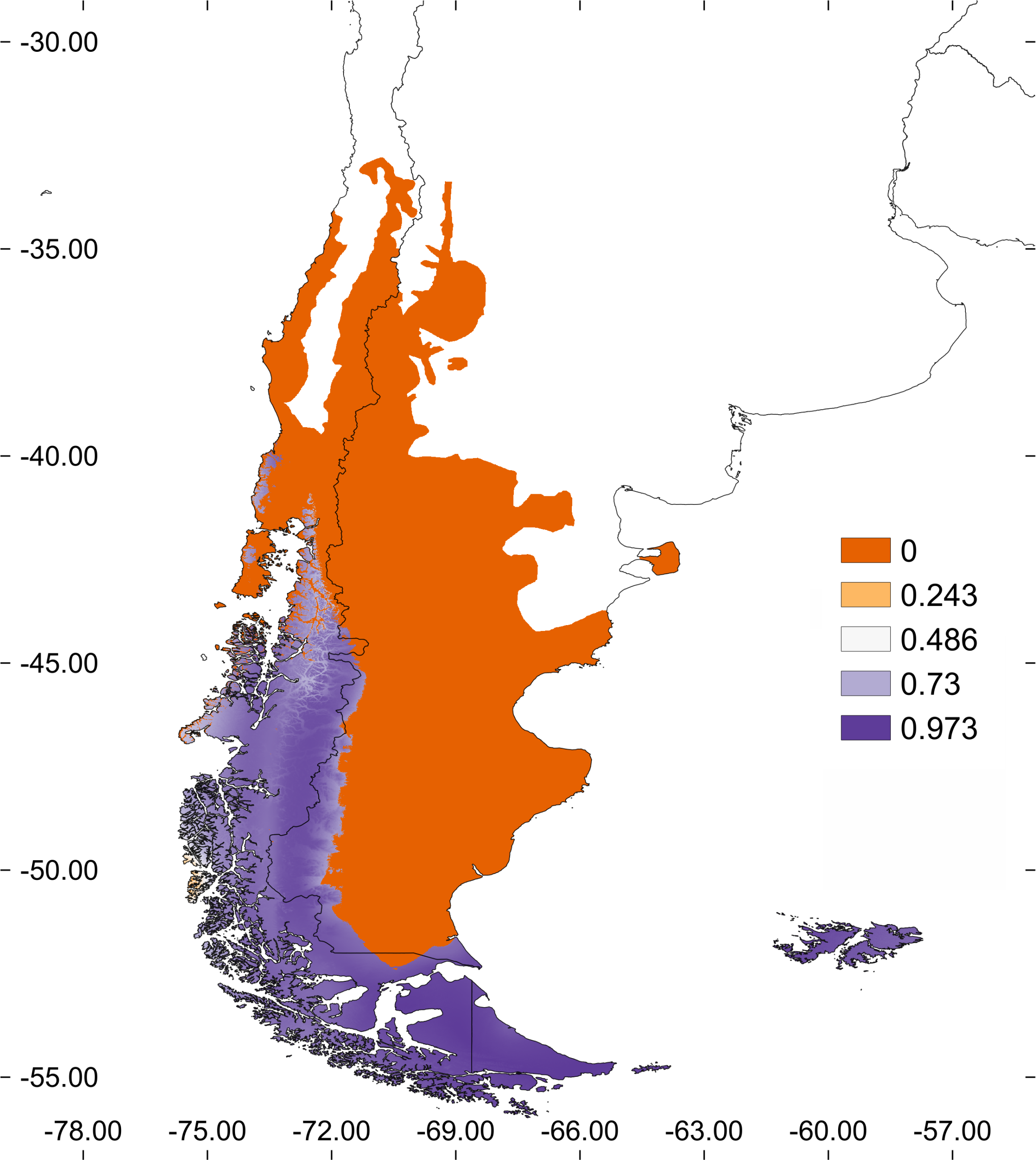

Supplement: Supplemental Information 5 — Areas with the most dissimilar variables conditions (i.e., where one or more environmental variables are outside the range present in the training data) are represented by zero value. These areas represents strict extrapolative areas so predictions in those areas should be treated with strong caution. Other values represent levels of similarity between the calibration area and the “G” transfer area. [file peerj-09-11614-s005.png]
