# Supplementary material for: Geographic distribution modeling and taxonomy of Stephadiscus lyratus (Cothouny in Gould, 1846) (Charopidae) reveal potential distributional areas of the species along the Patagonian Forests
Source: PeerJ. 2021 Jul 5;9:e11614. doi: 10.7717/peerj.11614 (PMC8265385; doi:10.7717/peerj.11614)
Supplement: Supplemental Information 6 [file peerj-09-11614-s006.docx]

***Species discovery***

*Helix lyrata* was discovered by Joseph Pitty Couthouy, the named conchologist of the "United States Exploring Expedition" to the South Pacific and Antarctic seas between 1838 and 1842, commanded by Charles Wilkes. The Expedition made base in Tierra del Fuego during 1838 and 1839, while Wilkes and part of the crew travel to Antarctica. Couthouy made careful and suggestive notes of all the interesting species and especially of the new ones, intending to amplify them upon his return (Johnson, 1964). Wilkes had antagonized with Couthouy during the trip when he refused to give Wilkes his notes and graphics from the trip. For this reason, Wilkes suspended him for "disobedience of orders" and order him home (Johnson, 1964). Gould (1852) said that "Cothouy's numerous notes were extremely valuable, especially those relating to the land snails...". Captain Couthouy had drawn up full notes on the external characters of the soft parts, habits, geographical distribution, and other essential points (Wyman, 1903). The collection of the Expedition upon arrival was sent initially to Peale's Museum in Philadelphia, later removed and sent to Washington, wherein 1856 was deposited at the Smithsonian Institution. When Couthouy returned to USA in 1840, he found that part of the material was lost or misplaced. Some of the collected specimens had been sent to prominent conchologists, as a favor, before any naturalist of the Expedition could work with them. Augustus Addison Gould was hired to study and publish all about the shells collected during the Expedition. He requested that the specimens were sent to Boston. Gould recognized Couthouy's work as a collector, as well as his notes describing species such as *Helix lyrata*. Then, the species description appeared in Gould's work on the shells of the Expedition (Gould, 1845-1848). Some of the shells collected during the Exploring Expedition are housed at the Museum of Comparative Zoology, Boston, while others are at the Smithsonian Institution. Duplicates and paratypes were exchanged with other Museums.
